# Repurposing Cefazolin-Avibactam for the Treatment of Drug Resistant *Mycobacterium tuberculosis*

**DOI:** 10.3389/fphar.2021.776969

**Published:** 2021-10-22

**Authors:** Shashikant Srivastava, Tawanda Gumbo, Tania Thomas

**Affiliations:** ^1^ Department of Pulmonary Immunology, University of Texas Health Science Centre, Tyler, TX, United States; ^2^ Department of Immunology, UT Southwestern Medical Center, Dallas, TX, United States; ^3^ Department of Pharmacy Practice, Texas Tech University Health Science Center, Dallas, TX, United States; ^4^ Praedicare Laboratories and Quantitative Preclinical & Clinical Sciences Department, Praedicare Inc., Dallas, TX, United States; ^5^ Division of Infectious Diseases and International Health, University of Virginia, Charlottesville, VA, United States

**Keywords:** beta-lactam, cephalosporin, avibactam, optimal dose, multidrug resistant tuberculosis

## Abstract

**Background:** While tuberculosis (TB) is curable and preventable, the most effective first-line antibiotics cannot kill multi-drug resistant (MDR) *Mycobacterium tuberculosis* (*Mtb*). Therefore, effective drugs are needed to combat MDR-TB, especially in children. Our objective was to repurpose cefazolin for MDR-TB treatment in children using principles of pharmacokinetic/pharmacodynamics (PK/PD).

**Methods:** Cefazolin minimum inhibitory concentration (MIC) was identified in 17 clinical *Mtb* strains, with and without combination of the β-lactamase inhibitor, avibactam. Next, dose-ranging studies were performed using the intracellular hollow fiber model of TB (HFS-TB) to identify the optimal cefazolin exposure. Monte Carlo experiments were then performed in 10,000 children for optimal dose identification based on cumulative fraction of response (CFR) and *Mtb* susceptibility breakpoint in three age-groups.

**Results:** Avibactam reduced the cefazolin MICs by five tube dilutions. Cefazolin-avibactam demonstrated maximal kill of 4.85 log_10_ CFU/mL in the intracellular HFS-TB over 28 days. The % time above MIC associated with maximal effect (EC_80_) was 46.76% (95% confidence interval: 43.04–50.49%) of dosing interval. For 100 mg/kg once or twice daily, the CFR was 8.46 and 61.39% in children <3 years with disseminated TB, 9.70 and 84.07% for 3–5 years-old children, and 17.20 and 76.13% for 12–15 years-old children. The PK/PD-derived susceptibility breakpoint was dose dependent at 1–2 mg/L.

**Conclusion:** Cefazolin-avibactam combination demonstrates efficacy against both drug susceptible and MDR-TB clinical strains in the HFS-TB and could potentially be used to treat children with tuberculosis. Clinical studies are warranted to validate our findings.

## Introduction

Despite significant progress in tuberculosis (TB) drug development, TB still ranks above HIV/AIDS in terms of the leading cause of death from a single infectious agent (World Health Organization, 2020). While TB is curable and preventable, emergence of multi-drug resistance renders many of the most effective first-line antibiotics ineffective. Globally, an estimated 500,000 developed rifampin resistant TB in 2019, including 78% who had multi-drug resistant TB (MDR-TB) (World Health Organization, 2020). Thus, the search for well-tolerated antimicrobials with efficacy against both drug-susceptible and MDR-TB strains continues. This problem is particularly pressing in children, especially those <3 years old who present with different disease pathology compared to adults: rightfully TB of children is considered a neglected disease. (Swaminathan and Rekha, 2010; Swaminathan and Ramachandran, 2015) Lately there is a renewed interest in repurposing antibiotics for treatment of TB and β-lactam class of antibiotics appear to be promising candidates. (Deshpande et al., 2016a; Ramón-García et al., 2016; Swaminathan et al., 2016; Deshpande et al., 2017; van Rijn et al., 2017; Srivastava et al., 2020a; Srivastava et al., 2020b)

We have found that several β-lactam antibiotics with β-lactamase inhibitor, including benzylpenicillin, and cephalosporins (ceftriaxone, ceftazidime, and cefdinir) have good efficacy against MDR-TB, in the hollow fiber model of TB (HFS-TB). (Deshpande et al., 2017; Deshpande et al., 2018; Srivastava et al., 2020b; Srivastava et al., 2021) Cefazolin is a first-generation cephalosporin, in use for various indications since the 1970s. (Kusaba, 2009) The population pharmacokinetics of cefazolin in critically ill children with methicillin-sensitive *Staphylococcus aureus* infection, as well as those undergoing surgery, have been published. (De Cock et al., 2017; Salvador et al., 2021) The drug is ∼80% protein bound. Almost 40 years of clinical use of cefazolin has resulted in ample evidence on efficacy against bacterial infections in children, and a good safety profile. (Kusaba, 2009) Here, we examined if cefazolin could also be effective for treatment of MDR-TB for children, using pharmacokinetics/pharmacodynamics studies in the HFS-TB for pediatric TB, followed by *in silico* dose finding clinical trial simulations. (Srivastava et al., 2011; Srivastava et al., 2016a; Deshpande et al., 2016b) Experiments were performed with or without combination of a β-lactamase inhibitor, avibactam, which we have previously found to improve the potency and efficacy of other β-lactams against *Mycobacterium tuberculosis* (*Mtb*). (Srivastava et al., 2020a)

## Materials and Methods

### Bacterial Strain, Cell Line and Supplies

*Mtb* laboratory strain H37Ra (ATCC#25177) and 17 clinical isolates (eight susceptible to rifampin and isoniazid and nine multidrug-resistant (MDR; resistant to both rifampin and isoniazid)) were used in the experiments. Culture conditions for the bacteria and the human-derived THP-1 cells (ATCC TIB-202) were the same as reported in our previous publications. (Srivastava et al., 2011; Srivastava et al., 2016a; Deshpande et al., 2016c; Srivastava et al., 2020a) Cefazolin and avibactam were purchased from BOC Sciences (New York, United States), isoniazid and rifampin from Sigma Aldrich (St. Louis, MO, United States), and mycobacterial growth indicator tube system (MGIT), related supplies and EpiCenter software were purchased from Becton Dickinson (Franklin Lakes, NJ, United States).

### Cefazolin MIC Distribution and Concentration-Response Studies

The bacterial culture media was Middlebrook 7H9 broth supplemented with 10% oleic acid-albumin-dextrose-catalase (OADC). We used the micro-broth dilution method (Woods et al., 2018) to determine cefazolin MICs for both *Mtb* H37Ra laboratory strain and the clinical strains. Avibactam was used at 15 mg/L final concentration, found to be optimal in *Mtb* studies published elsewhere. (Srivastava et al., 2020a) The cefazolin concentrations, in a two-fold dilution series, ranged from 0.25 to 256 mg/L. Prior to the experiments, cultures were grown to logarithmic phase followed by adjustment of turbidity to McFarland standard 0.5 and further 100-fold dilution to achieve ∼5 log_10_ CFU/mL bacterial density in the inoculum. Next, 180 μL of the inoculum was transferred to each well of 96-well tissue culture plates, prefilled with 20 μL of each drug concentration (10x). The cultures were incubated at 37°C for 7 days before MIC was read using an inverted mirror. The lowest drug concentration completely inhibiting visible growth of bacteria was recorded as the MIC. The experiments were performed twice with three replicates for each drug concentration.

The cefazolin concentration-response studies (at static concentration), with and without 15 mg/L avibactam, were performed in triplicate test tubes where the cefazolin concentration range was between 2 and 256 mg/L. Inoculum was prepared as described above, and cultures were co-incubated with each drug concentration at 37°C under shaking conditions for 7 days, followed by 10-fold serial dilutions and inoculation on Middlebrook 7H10 agar supplemented with 10% OADC agar (herein termed “agar”) for enumeration of the colony forming units (CFU). The CFUs were recorded after 21 days of incubation at 37°C. The experiment was performed twice with three-replicates for each drug concentration.

### Cefazolin Efficacy Against *Mycobacterium tuberculosis* Using the Intracellular Model of Hollow Fiber Model of Tuberculosis

In order to determine the optimal cefazolin exposure for *Mtb* kill, we used the intracellular HFS-TB model (Deshpande et al., 2016a; Srivastava et al., 2016a; Deshpande et al., 2017) mimicking human-like drug pharmacokinetics. (Alffenaar et al., 2020) The detailed descriptions of the model system have been previously published. (Deshpande et al., 2016a; Srivastava et al., 2016a; Deshpande et al., 2017) Briefly, 20 ml of *Mtb* H37Ra infected THP-1 monocytes (intended multiplicity of infection 1:1) were inoculated into the peripheral compartment of each of the 12 HFS-TB units. Cefazolin concentrations and daily dosing schedule were selected to mimic percentage of the time free (*f*) drug concentration remains above MIC (*f*%T_MIC_), ranging from 0 (non-treated control) to 100%T_MIC_. The treatment duration was 28 days. The drug was infused into the central compartment of the HFS-TB to achieve human-like cefazolin concentration-time profiles, mimicking 2 h half-life (t_1/2_). (Rattie and Ravin, 1975) Given the lack of available information on what %T_MIC_ of avibactam is required for efficacy of cefazolin, we added 15 mg/L avibactam to the circulating media (RPMI-1640 with 2% fetal bovine serum), thus a constant exposure to avibactam.

The concentration-time profile, of cefazolin and avibactam, achieved in each HFS-TB unit was validated by sampling the central compartments at pre-dose and 1, 3, 5, 11.5, 13, 15, and 23.5 h after the drug administration. The peripheral compartments were sampled every 7 days and samples were used to estimate bacterial burden using both colony forming unit (CFU) counts on solid agar and time-to-positivity (TTP) using the MGIT liquid culture system. (Hall et al., 2009; Srivastava et al., 2011; Srivastava et al., 2016a; Deshpande et al., 2016c; Srivastava et al., 2020a) To identify drug-resistant subpopulation, the samples were also cultured on agar supplemented with 3 times the MIC of cefazolin and 15 mg/L avibactam and incubated at 37°C under 5% CO_2_ for 28 days before the colonies were counted.

### Drug Concentration Assays

A multiplexed liquid chromatography tandem mass spectrometry (LC-MS/MS) method was used for all drug concentration measurements. The method used to measure avibactam concentration has been published previously and was used without any modification. (Srivastava et al., 2020a) For cefazolin, an LC-MS/MS assay was developed where calibrator, controls and internal standards (ceftazidime-d5) were included in each analytical run for quantitation. The LC-MS/MS analysis was performed using Waters Acquity UPLC connected to a Waters Xevo TQ mass spectrometer (Milford, MA). Compounds were detected using positive electrospray ionization in multiple reaction monitoring mode. The between day percentage coefficient of variation (%CV) for analysis of low and high controls of cefazolin was 3.1%. The intraday %CV was 5.1%. The lower limit of detection of this method was 0.75 mg/L.

### Pharmacokinetics/Pharmacodynamics Analysis

Drug pharmacokinetics in the HFS-TB were modeled using a one compartment model with first-order input and elimination, using ADAPT 5 and Phoenix WinNonLin (D'Argenio et al., 2009; Phoenix (2020)). The observed drug concentrations were then used to calculate the primary parameters that include peak concentration (C_max_) and area under the concentration-time curve (AUC_0-24_) as well as the secondary parameters including cefazolin half-life in the HFS-TB, peak concentration to MIC (C_max_/MIC) ratio, AUC_0-24_/MIC ratio, and the percentage of time that cefazolin concentration persisted above MIC (%T_MIC_). We used the inhibitory sigmoid E_max_ model using cefazolin concentration versus *Mtb* burden, to estimate the PK/PD exposure associated with 50% (EC_50_), or 80% (EC_80_), or 90% (EC_90_) of maximal bacterial kill (E_max_). For the HFS-TB studies, we identified the PK/PD index linked to *Mtb* kill using the same inhibitory sigmoid E_max_ model for CFU/mL versus exposure (%T_MIC_ or AUC_0-24_/MIC or C_max_/MIC) based on corrected Akaike Information Criteria (AICc) scores. (Akaike, 1974) GraphPad Prism (v8) was used for graphing.

### Monte Carlo Simulations for Dose Finding

Since the most important determinants of outcome in TB are drug concentrations achieved in patients, followed by MICs in terms of importance, and the child’s age (<3 years), we performed Monte Carlo experiments (MCE) that take pharmacokinetics, MIC, and age variability into account. (Swaminathan et al., 2016; Srivastava et al., 2016a; Chigutsa et al., 2015; Gumbo et al., 2014a; Gumbo et al., 2014b; Pasipanodya et al., 2018; Rockwood et al., 2017; Gumbo et al., 2015) The pharmacokinetic parameter estimates and covariance entered into the domain of input of subroutine PRIOR of ADAPT 5 (Biomedical Simulations Resources, University of Southern California) were from the literature (Table 1), which demonstrated clearance and volume of distribution varied with weight, based on ¾ fractal power laws, respectively. (De Cock et al., 2017; Salvador et al., 2021) In children <3 years who have disseminated disease and pulmonary disease that’s predominantly intracellular, blood concentrations were used for probability of target attainment probability (PTA) calculations. In preschoolers and teenagers, who develop adult-like pulmonary and cavitary disease, lung concentrations were used based on the literature, thus lung penetration and its variance were taken into account. We used the data published by Cole (Cole and Pung, 1977) in patients with bacterial pneumonia who had pleural and serum concentrations measured and performed compartmental pharmacokinetic modeling; pleural fluid concentrations were considered approximations of intrapulmonary concentrations in lung lesions and epithelial lining fluid. We also considered cefazolin protein binding based on the work of Vella-Brincat *et al* who demonstrated concentration-dependent protein binding, with less protein binding at high concentrations. (Vella-Brincat et al., 2007) In the MCE we examined the PTA for 100 mg/kg administered once every 24 h or twice every 24 h, as a half-hour infusion. The target PK/PD exposure was the cefazolin exposure that mediated 80% of E_max_ identified in the HFS-TB.

**TABLE 1:** Monte Carlo simulation model input and output for 10,000 children.

	In domain of input		Observed in 10,000 subject simulations	
	Median parameter estimate	%CV	Median parameter estimate	%CV
Children <3 years (disseminated TB)				
Clearance L/hr/kg	0.25	33.50	0.25	33.5
Intercompartmental clearance L/hr/kg	0.87	33.50	0.87	33.09
Central volume L/kg	0.31	31.80	0.31	31.66
Peripheral Volume L/kg	0.38	48.20	0.38	48.49
Children >3–5 years				
Clearance L/hr/kg		33.5	0.23	33.36
Intercompartmental clearance L/hr/kg		36.6	0.81	33.56
Central volume L/kg		31.8	0.29	31.65
Peripheral Volume L/kg		48.2	0.35	49.05
Children teenagers				
Clearance L/hr/kg	0.14	33.5	0.14	33.19
Intercompartmental clearance L/hr/kg	0.47	36.6	0.47	33.13
Central volume L/kg		31.8	0.17	31.66
Peripheral Volume L/kg		48.2	0.21	48.72

## Results

### Cefazolin-Avibactam MIC Distribution

**FIGURE 1:**
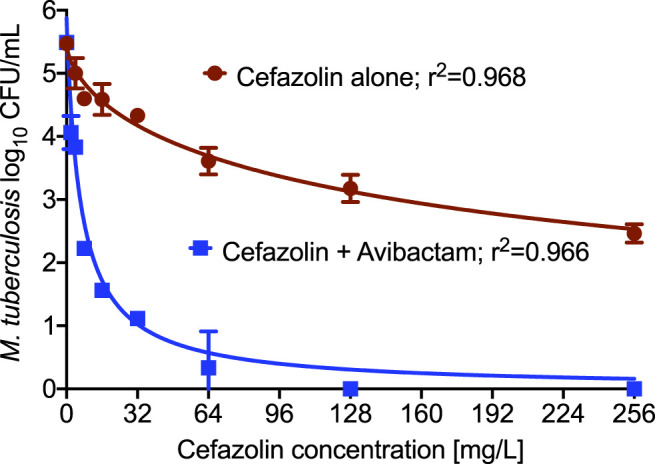
Cefazolin concentration response against *M. tuberculosis*. Addition of β-lactamase inhibitor, avibactam resulted in improved killing of *Mtb* as well as lower EC_50_ compared to the cefazolin alone.

MIC of the *Mtb* laboratory strain *Mtb* H37Ra was 64 mg/L for cefazolin alone, that decreased to 2 mg/L in the presence of 15 mg/L avibactam. Table 2 summarizes the cefazolin MICs against the 17 clinical strains determined in the presence of 15 mg/L avibactam, ranged from 2 to 8 mg/L. Figure 1 shows the results of the cefazolin concentration-response test-tube study with *Mtb* H37Ra with and without combination of 15 mg/L avibactam. The maximal *Mtb* kill (E_max_) by cefazolin alone was 3.01 log_10_ CFU/mL, and EC_50_ was calculated as 202.3 mg/L. On the other hand, in combination of 15 mg/L avibactam, E_max_ was 5.49 log_10_ CFU/mL, and EC_50_ was calculated as 6.44 mg/L. Thus, addition of avibactam resulted in improved cefazolin efficacy (E_max_) and potency (EC_50_) against *Mtb*.

**TABLE 2:** Cefazolin MIC distribution among *M. tuberculosis* clinical isolates. Isoniazid and rifampin were used at a single drug concentration to reconfirm the drug-susceptible or MDR-TB status. Cefazolin MIC_50_ (in combination with avibactam) for the clinical isolates was 2 mg/L, whereas MIC_90_ was calculated as 8 mg/L.

Clinical strain	Rifampin (1 mg/L)	Isoniazid (0.1 mg/L)	Cefazolin [with 15 mg/L avibactam]
1A	S	S	2
3A	S	S	2
6B	S	S	4
7A	S	S	2
8A	S	S	8
10B	S	S	2
18B	S	S	4
3D3	R	R	4
6C	R	R	8
7C4	R	R	2
9C	R	R	2
11D1	R	R	2
12D4	R	R	8
16D	R	R	2
18D3	R	R	2
20D2	R	R	2
14C3	R	R	2

S, sensitive; R, resistant.

### Cefazolin Dose-Effect in the Hollow Fiber Model of Tuberculosis

**FIGURE 2:**
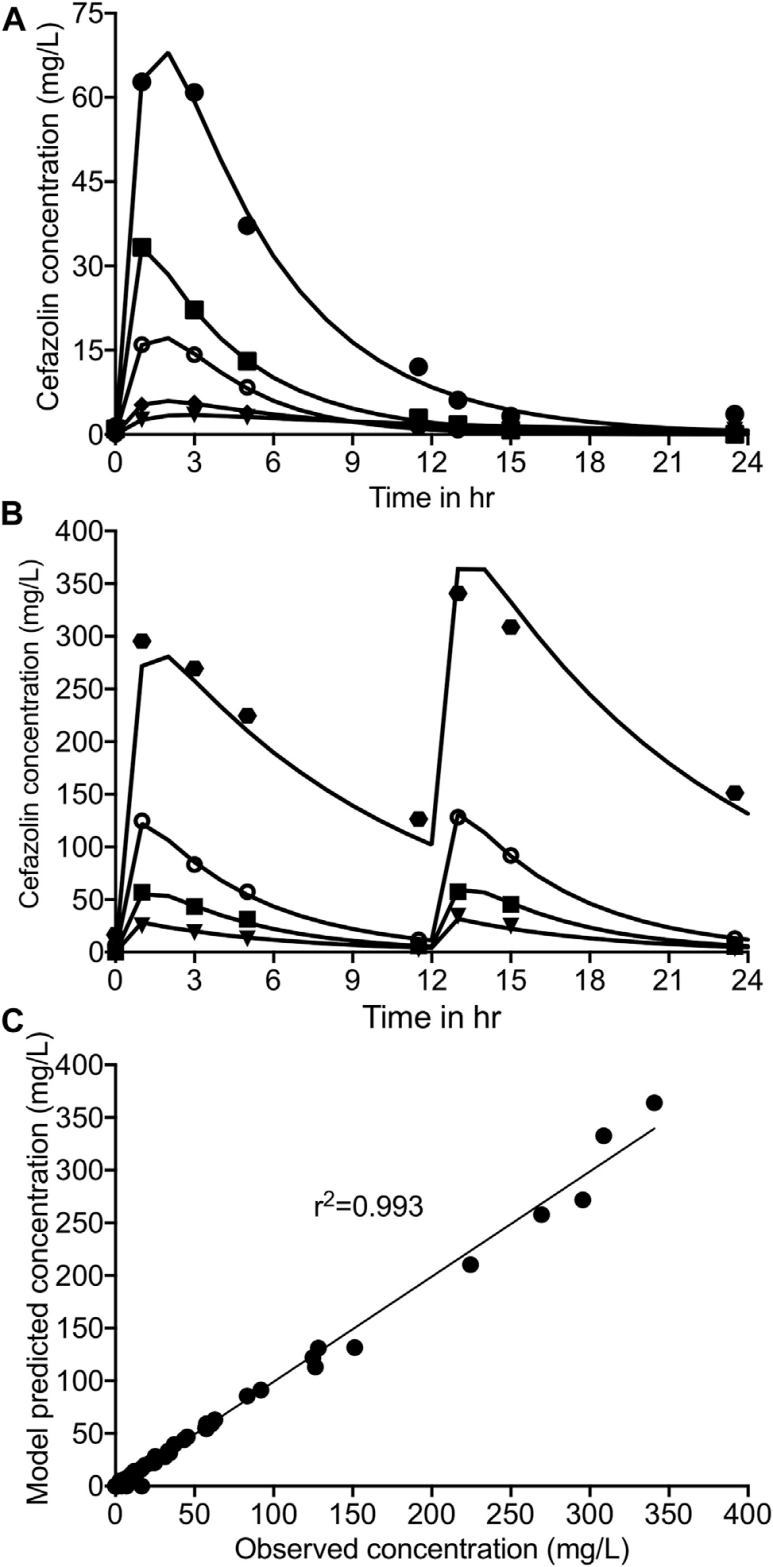
Cefazolin concentration-time profile in the HFS-TB. Symbols show the drug concentration measured in the HFS-TB, whereas solid line represent the model predicted concentrations. Cefazolin concentration time profile **(A)** with once daily dosing, **(B)** with twice-daily dosing schedule of cefazolin in the HFS-TB. **(C)** Model fit to show minimal bias between the cefazolin concentration measured in the HFS-TB vs the pharmacokinetics model predicted concentration.

Figure 2A shows the concentration-time profile of cefazolin achieved in the HFS-TB with once daily dosing schedule, whereas Figure 2B shows the same for twice-daily administration of cefazolin. The cefazolin half-life in the HFS-TB model was calculated as 3.21 ± 0.84 h, volume of distribution as 6.54 ± 3.08 L, and clearance as 1.26 ± 1.02 L/h. The regression between the pharmacokinetics modeled versus the HFS-TB measured cefazolin concentration for the model fit is shown in Figure 2C.

**FIGURE 3:**
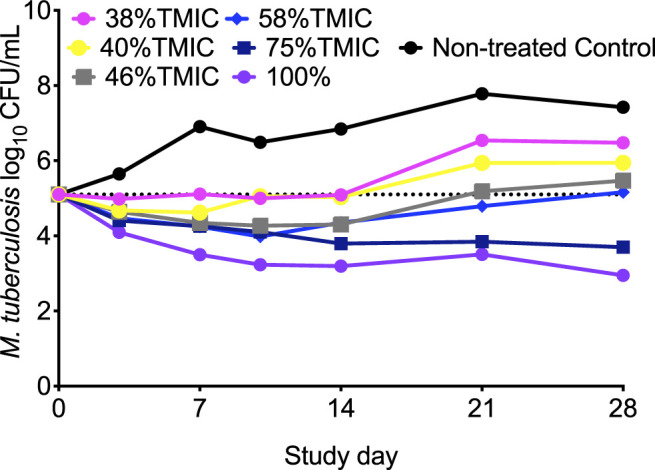
Cefazolin kill curves of intracellular *Mtb* in the HFS-TB. We pooled the cefazolin twice-daily exposures that were similar to once daily for clarity in the figure. Exposures ≥75% T_MIC_ kept the bacteria below stasis and the extent of kill was 2.52 log_10_ CFU/mL below stasis on day 28 of the study.

On day 28 of the intracellular HFS-TB study, the non-treated control systems showed the lowest number of viable THP-1 cells, whereas the number of viable cells increased from baseline (day 0) in the HFS-TB units treated with cefazolin doses achieving >75% %T_MIC_, indicating that the drug was indeed effective in controlling the intracellular *Mtb* burden (Supplementary Figure S1). Figure 3 show the kill curves with different cefazolin exposures in the HFS-TB. *Mtb* in non-treated control HFS-TB units grew from 5.10 log_10_ CFU/mL to 7.42 log_10_ CFU/mL in 28 days at a rate of 0.08 log_10_ CFU/mL/day. On day 28, while all cefazolin exposures had lower bacterial burden compared to the non-treated controls, the maximal kill of 4.85 log_10_ CFU/mL was observed with 100%T_MIC_ exposure. There was no cefazolin resistant *Mtb* colonies recorded on agar supplemented with 3X MIC cefazolin concentration and 15 mg/L avibactam, to any of the cefazolin dose tested in the HFS-TB. Supplementary Figure S2 shows the changes in the TTP (higher the TTP lower the *Mtb* bacterial burden in the HFS-TB) with each cefazolin exposure over the 28 days study period.

**FIGURE 4:**
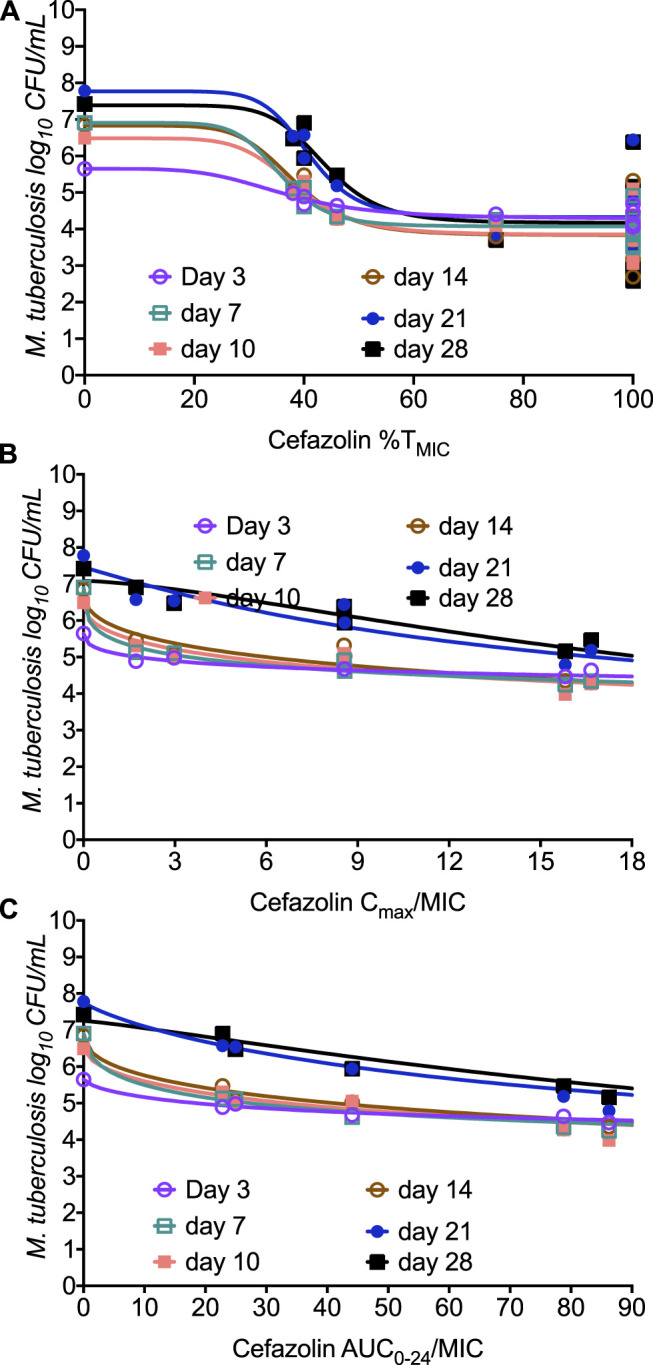
Relationship between *Mtb* burden and cefazolin exposure in the HFS-TB. The model fit for each sampling day is shown in the figure for **(A)** %T_MIC_, **(B)** C_max_/MIC, and **(C)** AUC_0-24_/MIC.

Figure 4 shows the results for the CFU/mL readouts where the 4-parameter inhibitory sigmoid model was used to determine the relationship between the cefazolin PK/PD parameters (%T_MIC_, or C_max_/MIC, or AUC_0-24_/MIC) and *Mtb* burden (Table 3). While best AICc score at each sampling time-point would seem to indicate C_max_/MIC linked-effect, the *r* ^2^ were not well separated. Moreover, the EC_50_ estimates for C_max_/MIC and AUC_0-24_/MIC varied widely from sampling day to sampling day (large % CV), while the EC_50_ and Hill-slope (H) for %T_MIC_ was more consistent and robust throughout the study with a %CV of only 7.65 and 22.9%, respectively. Thus, the EC_50_ for cefazolin was in a tight range of 38.9% of dosing interval (95% CI: 35.8–42.0%) throughout the study, and to an average EC_80_ of 46.76% (95% CI: 43.04–50.49%) of dosing interval over the entire study.

**TABLE 3:** Comparison of the PK/PD parameters for cefazolin’s efficacy against *M. tuberculosis*.

	%T_MIC_ EC_50_	% T_MIC_ H	AUC/MIC EC_50_	AUC/MIC H	C_max_/MIC EC_50_	C_max_/MIC H
Mean	38.9	7.53	96.2	0.731	24.4	0.718
Standard Deviation	2.98	1.72	58.6	0.262	18.6	0.458
Standard Error of Mean	1.22	0.703	23.9	0.107	7.59	0.187
Lower 95% CI of mean	35.8	5.72	34.7	0.456	4.87	0.238
Upper 95% CI of mean	42.0	9.34	158	1.01	43.9	1.20
Coefficient of variation	7.65%	22.9%	61.0%	35.8%	76.3%	63.7%

EC, effective concentration; H, Hill coefficient.

### The Relationship Between Cefazolin Serum and Intrapulmonary Concentrations

Our compartmental pharmacokinetic modeling of the cefazolin concentrations by Cole (Cole and Pung, 1977) using either the serum or pleural concentrations identified the pharmacokinetic parameter estimates shown in Table 4. The model-predicted versus observed concentrations had slopes of 0.99 (95% CI: 0.94–1.04) for serum and 0.989 (95% CI: 0.971–0.997) for pleural fluid, indicating minimal bias. This means that the clearances and half-life of intrapulmonary and serum concentrations were similar, and essentially parallel. The intrapulmonary/serum penetration ratio was variable and correlated with pleural drug penetration (Pearson correlation coefficient = 0.978; *p* < 0.0001); the relationship between pleural drug concentration and penetration ratio was: cefazolin concentration = 64.67*(Penetration ratio) + 0.04789 (*r* ^2^ = 0.957). This pattern is very similar to cefazolin protein binding, which we calculated from the published data to have a Pearson correlation coefficient = 0.856; *p* < 0.0001; unbound drug was 15% at low concentrations and 48% at high drug concentration. (Cole and Pung, 1977) This strongly suggests that cefazolin drug penetration into lung is driven by protein binding.

**TABLE 4:** ADAPT pharmacokinetic-model generated parameters using concentrations provided by Cole (Cole and Pung, 1977). In the study by Cole (Cole and Pung, 1977) ,cefazolin 1 g was administered intravenously. After 60 min of drug administration in nine patients, the reported C_max_ (mean ± Standard Deviation) was 65.65 + 29.65 mg/L in the serum and 12.45 + 5.75 mg/L in the pleural fluid (Cole and Pung, 1977).

	Serum estimate	% CV	Pleural estimate	% CV
Total clearance [L/hr]	1.441	64.9	1.469	85.03
Central volume [L]	1.525	89.26	1.71	102.88
Intercompartmental clearance [L/hr]	3.051	126.11	3.224	137.15
Peripheral volume [L]	3.368	105.08	2.696	125.06
Half-life [hr]	2.419	99.35	2.069	289.37
Model-predicted versus observed concentration *r* ^2^	0.996		0.999	

### Monte Carlo Simulations for Dose Finding in Three Children Age Groups

**FIGURE 5:**
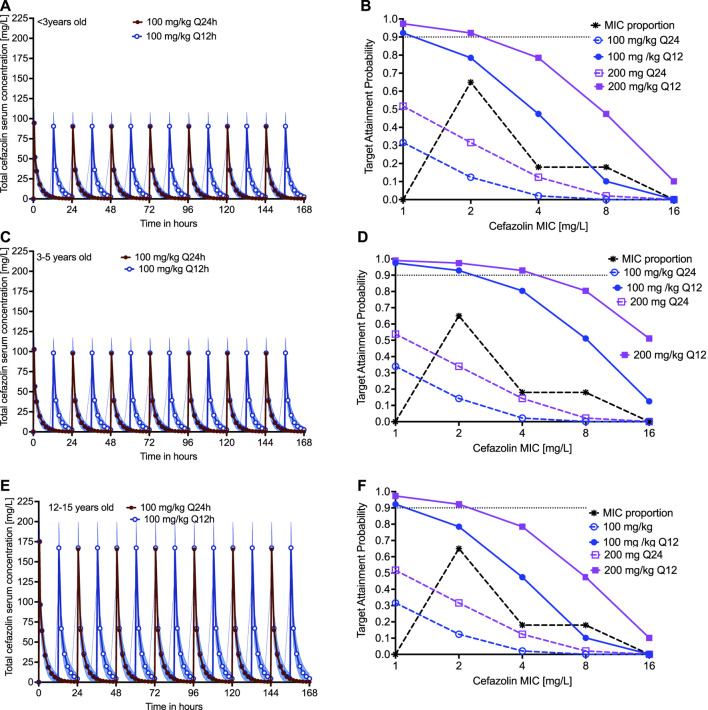
Cefazolin clinical dose and susceptibility breakpoint. The probability of attaining the exposure target with a given dose decrease with increase in the MIC of Mtb. The 7 days simulated PK profile with 100 mg/kg and 200 mg/kg dose is shown for children **(A)** <3 years-old, **(C)** 3–5 years-old, and **(E)** 12–15 years-old. The corresponding probability of target attainment, summated over the MIC in clinical strains of *Mtb*, in these three different age groups, for two different clinical doses administered as once or twice daily is shown in **(B)**, **(D)**, and **(F)**. We proposed the cefazolin susceptibility breakpoint for *Mtb* as 1–2 mg/L.

For children <3 yearsold, the simulated 7 days concentration time profiles (total drug) in the blood are shown in Figure 5A, for the dose of 100 mg/kg infused over 30 min either once a day or twice a day. The PTA in children <3 years-old, in which we took into account protein binding, was as shown in Figure 5B, which includes a dose of 200 mg/kg. The CFR in children <3 years-old was 8.46 and 61.39% in children treated with 100 mg once or twice a day, respectively, and was 25.01 and 82.63% in children treated with 200 mg once or twice a day, respectively. For children 3–5 years old, the simulated 7 days total cefazolin concentration time profiles in the blood are shown in Figure 5C; the PTA was as shown in Figure 5D. The CFR was 9.70 and 84.07% in children treated with 100 mg once or twice a day and was 25.08 and 94.45% in children treated with 200 mg once or twice a day, respectively. Teenagers develop pulmonary disease that resembles that in adults, and thus we used pleural concentration penetration ratios, that vary depending on cefazolin concentration. The simulated 7 days total cefazolin concentration time profiles in the blood of teenagers are shown in Figure 5E, and the PTA was as shown in Figure 5F. The CFR was 17.20 and 76.13% in teenagers treated with 100 mg once or twice a day and was 34.82 and 91.03% in children treated with 200 mg once or twice a day, respectively. Thus, twice a day dosing was superior to once-a-day dose, which is not surprising given the half-life of cefazolin. At the most optimistic, all doses and dosing strategies did poorly by an MIC of 4 mg/L; however, in most age groups and doses the PK/PD derived breakpoint was dose dependent at 1–2 mg/L.

## Discussion

The emergence of drug resistance during the therapy is one of the reasons the global targets (World Health Organization, 2020) to eliminate the TB disease is not achieved. There is an urgent need of developing new and effective treatment regimens that can prevent the emergence of resistance during therapy. One goal is that the new regimens should be equally effective against both drug-susceptible and drug-resistant strains of *Mtb*. Our group has developed a stepwise programmatic approach (Srivastava et al., 2016b) to screen drugs from different classes that are already in clinical use for other indications, and repurpose them for the treatment of TB, including MDR-TB.

In the present study, we studied cefazolin, which is used to treat many bacterial infections. Cefazolin provides excellent coverage against Gram-positive bacteria as well as has modest coverage against some Gram-negative bacterial pathogens. In the present study, first, we showed that the extent of *Mtb* kill with cefazolin alone was comparable to several of the first-line anti-TB drugs, and when combined with the β-lactamase inhibitor avibactam, the efficacy surpassed the standard anti-TB drugs. Second, combination with avibactam resulted in a 32-fold lower MIC of the standard laboratory strain. The MIC distribution among the MDR-TB clinical strains demonstrates that cefazolin has the potential to be developed for the treatment of TB.

Third, β-lactam antibiotics require a maximal duration of exposure of the unbound or free concentration of the drug at a level that should be greater than the MIC of the infecting organism. This is known as time-dependent killing, and consistent with the class, cefazolin’s efficacy is no different against Gram-positive and Gram-negative bacteria. In the HFS-TB model, we found that the PK/PD index linked to cefazolin efficacy against *Mtb* was *f*%T_MIC_ and the optimal exposure target or EC_80_ was determined as 46.76*f*%T_MIC_.

Fourth, our clinical trial simulations show the likely efficacy of cefazolin dose of 100 mg or 200 mg twice daily in children. The cumulative fraction of response of 200 mg twice daily in children under 3 years was 82.63%, for children between 3–5 years was 94.45%, and in teenage children was 91.03%. To put these findings in the clinical context, for other bacterial infections, frequent and higher doses of cefazolin are common: 1–2 g administered every 6–8 h (i.e., 4–6 g daily) is recommended to treat moderate to severe infections. (Author Anonymous) In the setting of severe *Staphylococcus aureus* infections, a 3 g dose administered twice daily for 6 weeks has been used with a reassuring safety profile. (Birrell and Fuller, 2019) This means that the clinical dose we propose to use for the treatment of MDR-TB will likely be safe (Kusaba, 2009), however, given the length of TB treatment, the effect of long-term cefazolin administration needs to be determined.

While the present study report efficacy of cefazolin as well as cefazolin-avibactam combination against MDR-TB, the study has its limitations. The *in vitro* findings may not always translate to the clinical setting; therefore, caution should be used while testing these doses to treat patients with TB. Moreover, the preclinical models cannot predict the risk of toxicity with double β-lactam (as the only commercial source of avibactam is a combination of ceftazidime-avibactam, AVYCAZ (2015)) during the prolonged TB treatment duration. Finally, while injectable drugs are used for the treatment of highly drug-resistant TB, recent WHO recommendations have discouraged their use. (Reuter et al., 2017; Nahid et al., 2019)

To conclude, the cefazolin-avibactam combination has efficacy against both drug-susceptible and MDR-TB clinical strains, the PK/PD index linked to efficacy was 46.76*f*%T_MIC_, and 200 mg/kg dose was predicted to achieve the optimal exposure target in >90% of the children to treat drug-resistant TB. Clinical validation of these pre-clinical findings remains to be done.

## Data Availability

The original contributions presented in the study are included in the article/Supplementary Material, further inquiries can be directed to the corresponding author.
