# Supplementary figures and images for: Repurposing Cefazolin-Avibactam for the Treatment of Drug Resistant Mycobacterium tuberculosis
Source: Front Pharmacol. 2021 Oct 22;12:776969. doi: 10.3389/fphar.2021.776969 (PMC8569112; doi:10.3389/fphar.2021.776969)

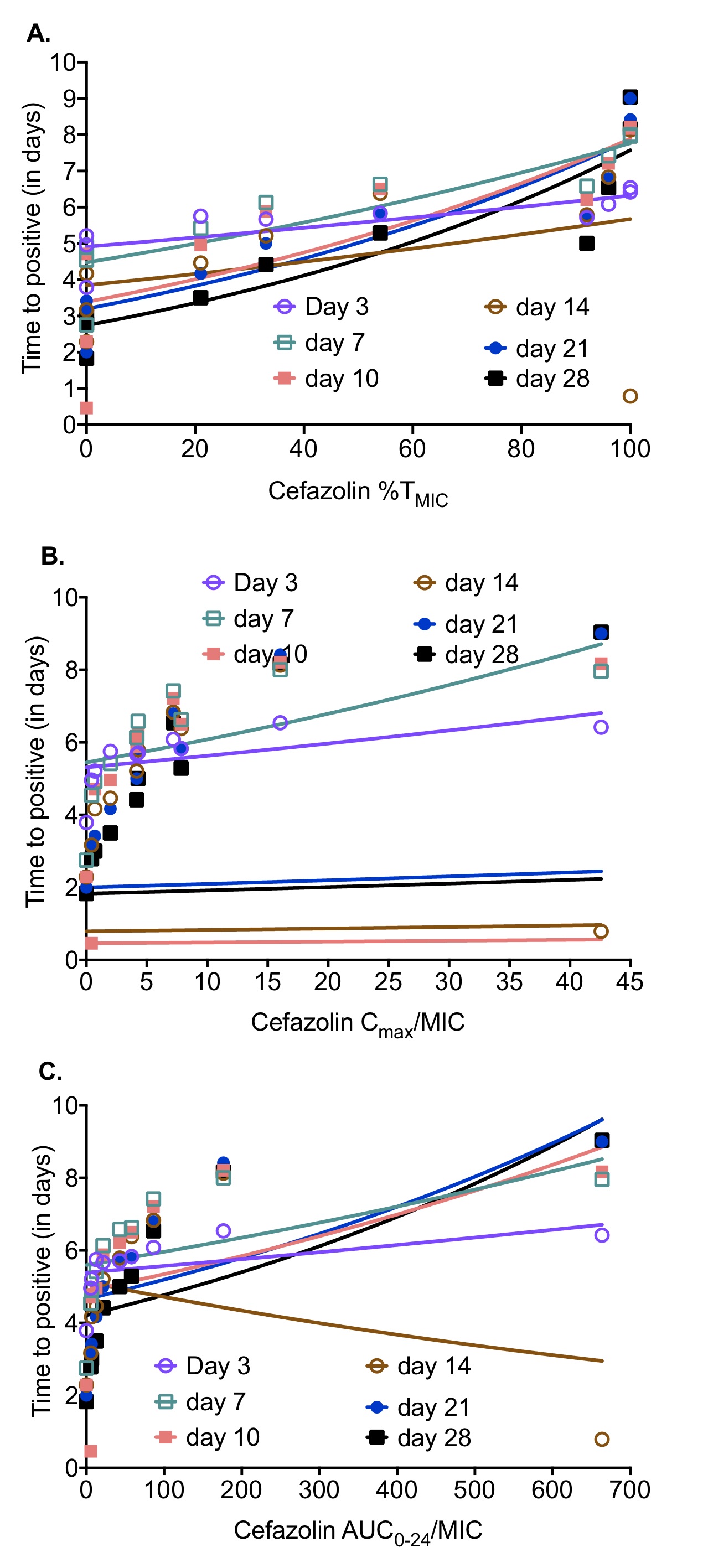

Supplement: Supplementary file 1 [file Image3.JPEG]

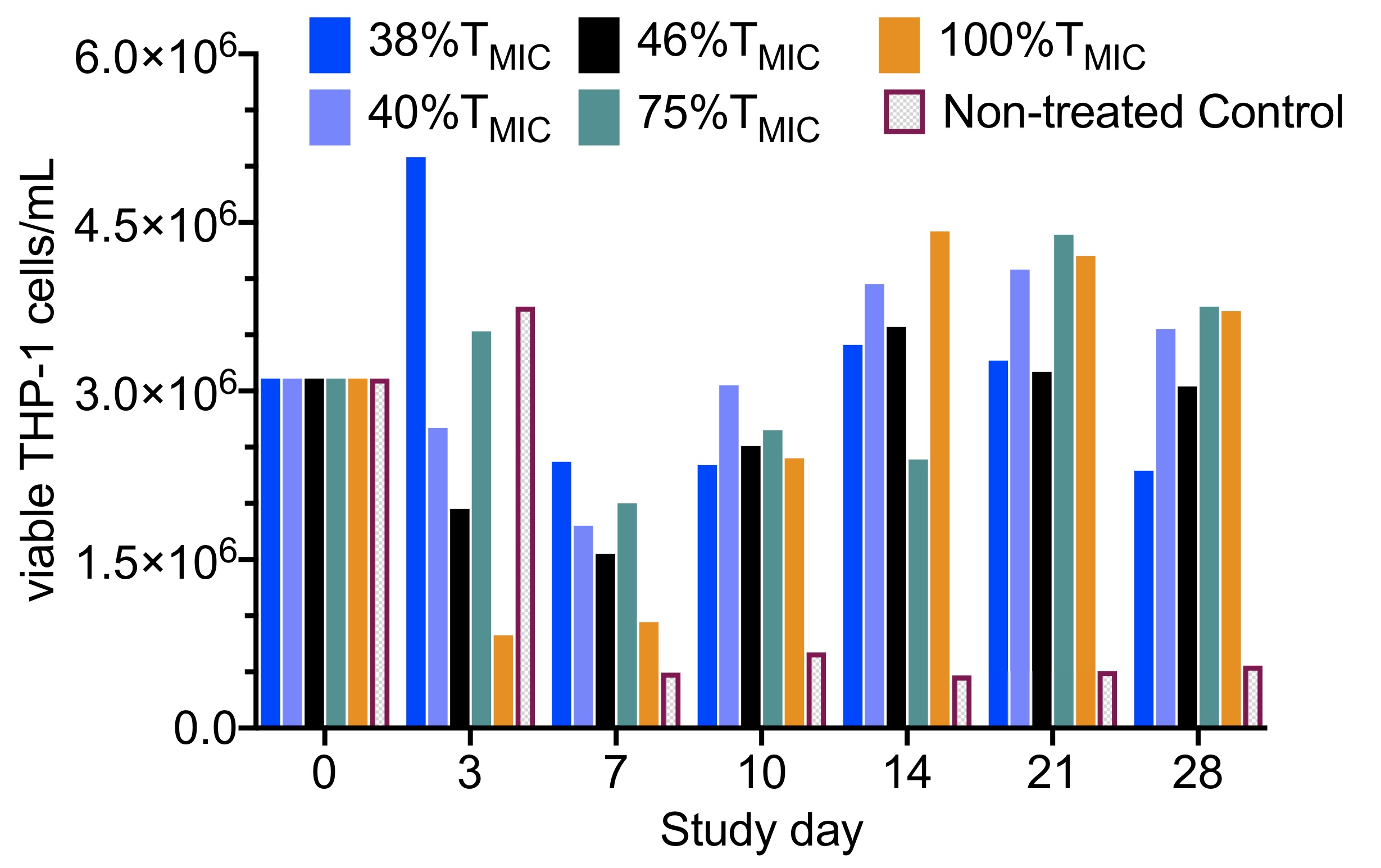

Supplement: Supplementary file 2 [file Image1.JPEG]

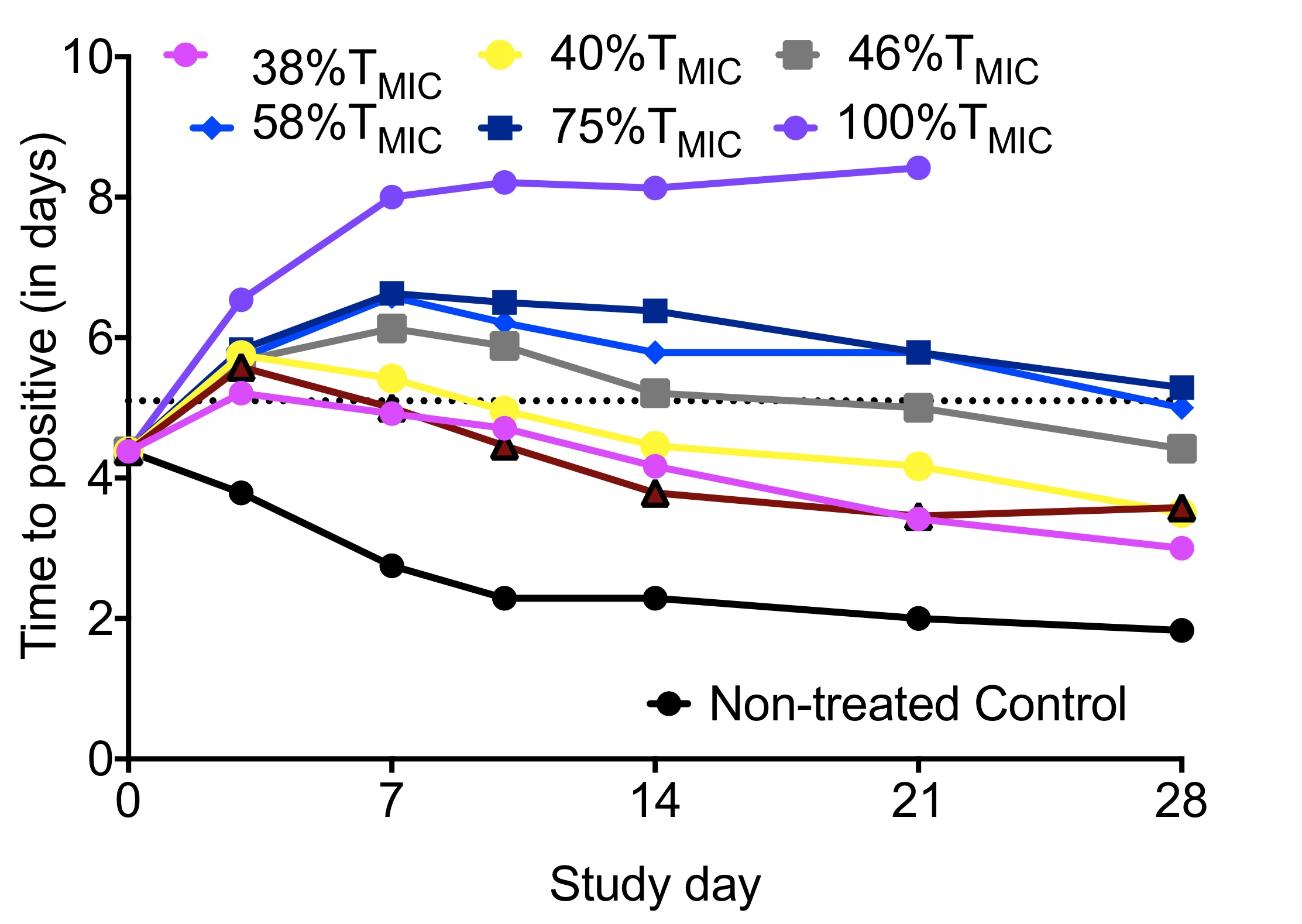

Supplement: Supplementary file 3 [file Image2.JPEG]
